# A Comparative Uptake Study of Multiplexed PET Tracers in Mice with Turpentine-Induced Inflammation

**DOI:** 10.3390/molecules171213948

**Published:** 2012-11-26

**Authors:** Tingting Huang, Hongliang Wang, Ganghua Tang, Xiang Liang, Dahong Nie, Chang Yi, Kening Wu

**Affiliations:** Department of Nuclear Medicine, The First Affiliated Hospital, Sun Yat-Sen University, Guangzhou 510080, China; E-Mails: mandyhtt@163.com (T.H.); hongliang0812@163.com (H.W.); liangx2003@126.com (X.L.); niedahong@126.com (D.N.); ares4310@yahoo.com.cn (C.Y.); gtanggroup@yahoo.com.cn (K.W.)

**Keywords:** inflammation, PET imaging, turpentine oil, [^18^F]FDG, multiple PET tracers

## Abstract

The potential value of multiplexed positron emission tomography (PET) tracers in mice with turpentine-induced inflammation was evaluated and compared with 2-[^18^F]fluoro-2-deoxy-D-glucose ([^18^F]FDG) for glucose metabolism imaging. These PET tracers included [^18^F]fluoromethylcholine ([^18^F]FCH) for choline metabolism imaging, (S-[^11^C]methyl)-D-cysteine ([^11^C]DMCYS) for amino acid metabolism imaging, [^11^C]bis(zinc(II)-dipicolylamine) ([^11^C]DPA-Zn^2+^) for apoptosis imaging, 2-(4-*N*-[^11^C]-methylaminophenyl)-6-hydroxybenzothiazole ([^11^C]PIB) for β amyloid binding imaging, and [^18^F]fluoride (^18^F^−^) for bone metabolism imaging. In mice with turpentine-induced inflammation mice, the biodistribution of all the tracers mentioned above at 5, 15, 30, 45, and 60 min postinjection was determined. Also, the time-course curves of the tracer uptake ratios for inflammatory thigh muscle (IM) to normal uninflammatory thigh muscle (NM), IM to blood (BL), IM to brain (BR), and IM to liver (LI) were acquired, respectively. Moreover, PET imaging with the tracers within 60 min postinjection on a clinical PET/CT scanner was also conducted. [^18^F]FDG and ^18^F^−^ showed relatively higher uptake ratios for IM to NM, IM to BL, IM to BR, and IM to LI than [^18^F]FCH, [^11^C]DPA-Zn^2+^, [^11^C]DMCYS and [^11^C]PIB, which were highly consistent with the results delineated in PET images. The results demonstrate that ^18^F^−^ seems to be a potential PET tracer for inflammation imaging. [^18^F]FCH and [^11^C]DMCYS, with lower accumulation in inflammatory tissue than [^18^F]FDG, are not good PET tracers for inflammation imaging. As a promising inflammatory tracer, the chemical structure of [^11^C]DPA-Zn^2+^ needs to be further optimized.

## 1. Introduction

2-[^18^F]Fluoro-2-deoxy-D-glucose ([^18^F]FDG) has been widely used as a glucose metabolism tracer for the diagnosis of various diseases and treatment evaluation in positron emission tomography (PET) imaging due to its increased glucose utilization [1,2,3,4]. However, [^18^F]FDG uptake is not specific to tumors and it frequently accumulates in benign lesions. Various forms of inflammatory lesions also take up [^18^F]FDG, which is a major cause of false-positive results in PET diagnosis [5,6]. The feature of high [^18^F]FDG uptake in inflammatory lesions has even been used for the detection of inflammation [5], making [^18^F]FDG-PET become a simple *in vivo* method to validate inflammatory models’ reality. 

Currently, a wide variety of radiotracers have been used for imaging of inflammation. However, only a few agents are used for inflammation imaging. These tracers include [^18^F]FDG, ^99m^Tc- or ^111^In-labeled autologous white blood cells (WBC) or proteins, ^99m^Tc-labeled bisphosphonates or nanocolloids, and [^67^Ga]citrate [7]. Though [^18^F]FDG PET was the first molecular imaging technique widely used for brain, cardiac, or cancer imaging in clinical settings [8], it is difficult to discriminate tumors and inflammatory diseases. The other tracers also have their limitations. Thus, so far there are no specific PET tracers for inflammation imaging in clinic. On the other hand, inflammation is a complex process, which involves many factors which can change over time and affect PET tracer uptake, including increased perfusion, infiltration of immune cells and stress responses of tissue cells [9]. As a result, amino acid metabolism, choline metabolism, apoptosis, neuronal degeneration, and increased blood flow can be also associated with inflammatory response. Therefore, it is very important for us to select a moderate PET tracer for inflammation imaging from among the existing multiple tracers related to biomarkers of these processes. 

[^18^F]Fluoride (^18^F^−^) as a bone metabolism imaging agent [10], [^18^F]fluoromethylcholine ([^18^F]FCH) as a choline metabolism tracer [11], and 2-(4-*N*-[^11^C]methylaminophenyl)-6-hydroxybenzothiazole ([^11^C]PIB) as a *β*-amyloid-targeted tracer [12], have been used for clinical PET imaging for several years. Recently, we have developed [^11^C]bis(zinc(II)-dipicolylamine) ([^11^C]DPA-Zn^2+^) as a new phospholipid (PS)-targeted apoptotic small-molecule tracer [13] and S-[^11^C]methyl)-D-cysteine ([^11^C]DMCYS) as a novel amino acid metabolism tracer. Though high uptake of [^18^F]FCH in inflammatory tissues was found [11], inflammation imaging with ^18^F^−^, [^11^C]PIB, [^11^C]DPA-Zn^2+^, and [^11^C]DMCYS was not reported. Perhaps one of these tracers can provide novel data and expect to improve the differentiating accuracy of tumor from inflammation. In this study, we estimate potential application of the multiple tracers (^18^F^-^, [^18^F]FCH, [^11^C]PIB, [^11^C]DPA-Zn^2+^, and [^11^C]DMCYS) in PET imaging of mice with turpentine-induced inflammation and compared them with [^18^F]FDG. 

## 2. Results and Discussion

### 2.1. PET Tracers

The uncorrected radiochemical yield of [^11^C]DMCYS from ^11^CH_3_I was more than 50% and the total synthesis time from ^11^CO_2_ was about 12 min. The retention time was approximately 3.0–4.0 min for [^11^C]DMCYS, less than 1.5 min for D-cysteine, and approximately 14.6 min for ^11^CH_3_I. The uncorrected radiochemical yield of [^11^C]DPA-Zn^2+^ was 8%–12% within 40 min based on [^11^C]methyl triflate. The specific radioactivity of [^11^C]DPA-Zn^2+^ and [^11^C]DMCYS were 3.5–5.4 and 3.6–5.0 GBq/μmol, respectively. All tracers were produced in our lab, with radiochemical purity > 95%.

### 2.2. Biodistribution of Multiple Tracers

The uptakes of [^18^F]FDG, ^18^F^−^, [^18^F]FCH, [^11^C]PIB, [^11^C]DPA-Zn^2+^ and [^11^C]DMCYS in inflammatory mice at 60 min postinjection are shown in Table 1. [^18^F]FDG, ^18^F^−^, [^18^F]FCH, [^11^C]DPA-Zn^2+^ and [^11^C]DMCYS showed physiologically high uptakes in heart (12.5±2.23% ID/g), femur (21.97 ± 8.02% ID/g), liver (20.51 ± 12.24% ID/g), liver (3.31 ± 1.09% ID/g), and pancreas (4.17 ± 1.54% ID/g), respectively. [^11^C]PIB accumulation in various organs was significantly lower than those of the other tracers. The radioactivity of brain and heart in [^18^F]FDG group, liver in [^18^F]FCH group, and femur in ^18^F^−^ group was higher than that in the other PET tracer groups (*p* < 0.05). The uptakes of [^18^F]FDG and ^18^F^−^ in inflammatory tissues were approximately 2.7-fold higher than those of [^18^F]FCH and [^11^C]DMCYS, 4.0-fold higher than those of [^11^C]DPA-Zn^2+^ and 14.3-fold higher than those of [^11^C]PIB, respectively.

The ^18^F^−^ uptake ratios of inflammation-to-muscle (IM/NM, 11.23 ± 2.32) was the highest among all the PET tracers studied (*p* < 0.01) (Table 1) and [^11^C]PIB had the lowest uptake ratios. The inflammation-to-blood (IM/BL) and IM/NM ratios of [^11^C]DPA-Zn^2+^ and [^11^C]DMCYS were relatively low.

### 2.3. Inflammation PET Imaging with Multiple Tracers

**Figure 1:**
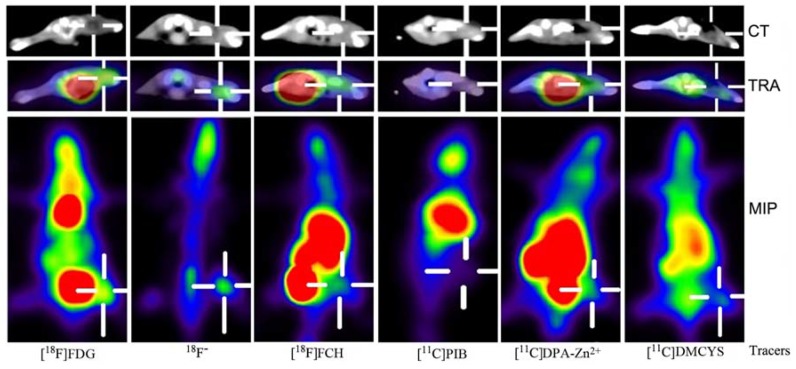
The transverse (TRA) CT and PET/CT fusion images, and the coronal maximum intensity projection (MIP) images in IM (cross-shaped lines) with multiple tracers ([^18^F]FDG, ^18^F^−^, [^18^F]FCH, [^11^C]PIB, [^11^C]DPA-Zn^2+^ and [^11^C]DMCYS) at 60 min post-injection. The high uptake of ^18^F^−^ and [^18^F]FDG was observed. In addition, the moderate uptake in [^18^F]FCH, [^11^C]DPA-Zn^2+^ and [^11^C]DMCYS, and almost no uptake of [^11^C]PIB in IM were demonstrated. Moreover, the intense uptake of [^18^F]FCH and [^11^C]DPA-Zn^2+^ in abdomen was found. The background activity of ^18^F^−^ was extremely low.

Figure 1 demonstrates PET imaging of inflammation with six tracers ([^18^F]FDG, ^18^F^−^, [^18^F]FCH, [^11^C]PIB, [^11^C]DPA-Zn^2+^, and [^11^C]DMCYS) after 60 min post-injection. The radioactivities of the tracers were localized in the right thigh inflammatory muscle in distinct levels. The high uptake of ^18^F^−^ and [^18^F]FDG, and the moderate uptake of [^18^F]FCH, [^11^C]DPA-Zn^2+^, and [^11^C]DMCYS were observed in PET images. Furthermore, almost no uptake of [^11^C]PIB in IM was found. The coronary maximum intensity projection (MIP) images demonstrated that heart uptake of [^18^F]FDG, abdomen uptake of [^18^F]FCH and [^11^C]DPA-Zn^2+^ was very high. All these PET imaging results were extremely consistent with the biodistribution results reported above.

### 2.4. Tracer Uptake Ratio-Time Curves

**Figure 2:**
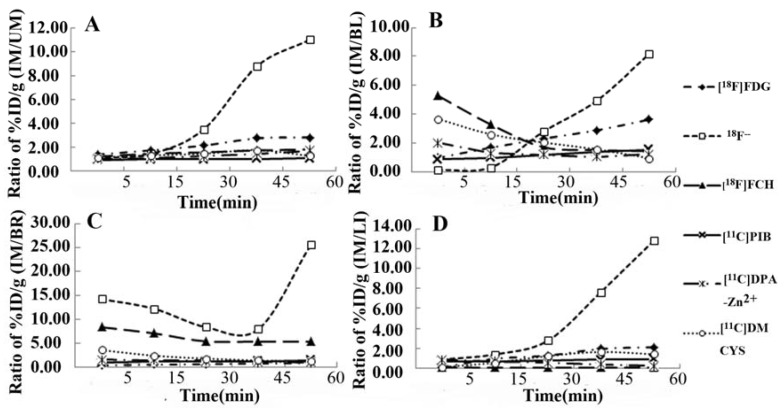
Time-uptake ratios curves of six PET tracers including IM/NM (**A**), IM/BL (**B**), IM/BR (**C**) and IM/LI (**D**) at different time. The ^18^F^−^ uptake ratios was progressively elevated toward 60 min after tracer administration, clearly contrasting with the other five tracers.

Tracers’ uptake ratios of IM/NM, IM/BL, IM/BR, and IM/LI are shown in Figure 2. Except for ^18^F^−^, most tracers’ uptake ratios gradually increased up to a constant value until 45 min and 60 min post-injection. Compared with the other five PET tracers, ^18^F^-^ exhibited the highest uptake ratios of IM/NM, IM/BL, IM/BR, and IM/LI at most observed time points, followed by [^18^F]FDG. The other tracers demonstrated low uptake ratios.

### 2.5. Histopathologic Findings of Inflammation

**Figure 3:**
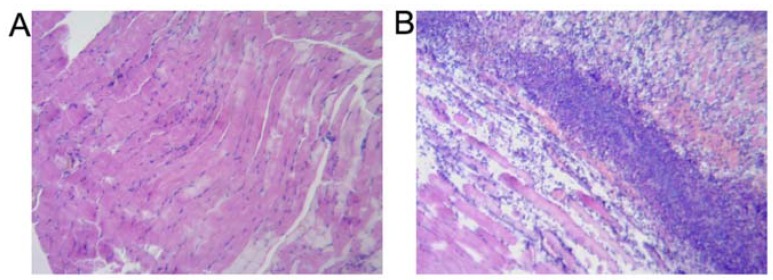
Microscopic observation of the three days’ inoculated right thigh inflammatory tissue (**B**) and the left thigh normal tissue (**A**). Microscopically, there were many inflammatory cells infiltrating among striated muscle in the HE staining (**B**), and the normal striated muscle tissue was also displayed (**A**) (all specimens were stained by hematoxylin and eosin and magnified by ×100).

A typical turpentine-induced inflammatory tissue observed by HE staining is shown in Figure 3. Three days after inoculation, there were numerous inflammatory cells infiltrating among striated muscle in the HE staining (Figure 3B). On the other hand, in the contralateral normal thigh muscle, normal striated muscle tissue was displayed (Figure 3A).

[^18^F]FDG has been extensively used in tumor, heart, and brain PET imaging. [^18^F]FDG accumulated in the inflammatory tissues, presumably explained by the active uptake of [^18^F]FDG by phagocytes within the inflammatory tissues or by granulation tissue surrounding the inflammatory tissues [14]. In our experiments, inflammatory tissue 3 days after turpentine-treatment showed chronic inflammation, which was characterized by fibroblast proliferation and neovascularization with mononuclear cell infiltration (macrophages, lymphocytes, and plasma cells). [^18^F]FDG uptake in turpentine-induced inflammatory tissue was 2.93-fold higher than that seen in contralateral healthy muscle. These results were consistent with those of the sterile rats’ inflammatory models [15]. These mice inflammatory models confirmed by histopathological analysis could be also used to evaluate the potential PET tracers for inflammation imaging. In recent years, multi-tracer PET imaging has been developed for wide use in clinical diagnosis and therapy evaluation. In this work, evaluation of the multiple PET tracers ([^18^F]FCH, [^11^C]DPA-Zn^2+^, [^11^C]DMCYS, [^11^C]PIB and ^18^F^−^) for turpentine-induced inflammation imaging was performed in comparison with [^18^F]FDG. 

^18^F^−^ is a well-established positron-emitting bone-seeking agent, and its uptake reflects both blood flow and remodeling of bone [16,17,18]. In our experiments, the histological examination of turpentine-induced inflammatory tissue on Day 3–4 showed the characteristic features of chronic inflammation with fibroblasts and vascular proliferation and macrophage infiltration [14]. Like [^18^F]FDG, high uptake of ^18^F^−^ was also found in turpentine-inoculated IM in our work, which was consistent with the uptake of ^18^F^−^ in periprosthetic infection of aseptic loosening cases reported by Choe *et al.* [17]. Kobayashi *et al.* [18] have also reported significant differences in the ^18^F^−^ uptake between aseptic and septic loosening total hip arthroplasty (THA) cases could be caused by the increased local blood flow due to acute inflammation following infection and by the severity of the loosening in infectious loosening cases. Thus, we can speculate that the proposed mechanism of the uptake of ^18^F^−^ in the aseptic inflammatory lesion is due to increased blood flow in the inflammatory tissue, together with enhancing permeability of capillary blood vessels, resulting in ^18^F-fluoride being incorporated into macrophages or a leakage of ^18^F^-^ fluid into the extravascular space at the chronic stage of inflammation. In this study, ^18^F^-^ is regarded as a potential inflammation-localizing agent because it demonstrated the best inflammation to normal tissue uptake ratios. 

Choline is transported into cells by specific mechanisms and phosphorylated by choline kinase, and then it is metabolised to phosphatidylcholine and incorporated into the cell membrane. [^18^F]FCH as a choline analogue is a lipid metabolism tracer and can be used for the detection of prostate cancer [19] and inflammation lesions [20,21]. However, the previous studies showed contradictory results for inflammation imaging. Some researchers found that high uptake of [^18^F]FCH in bacterial inflammation could limit the specificity of [^18^F]FCH for tumor detection [20], while other researchers observed low uptake of [^18^F]FCH in turpentine-induced aseptic inflammation in rats [22]. Avid [^18^F]FCH accumulation in bacterial inflammatory tissue could be explained by a major part of the [^18^F]FCH being incorporated into macrophages at the chronic stage of inflammation, indicating an upregulation of choline kinase in these inflammatory cells [22]. However, these paradoxical results could be contributed to by the different inflammatory models used. In our experimental study, relatively low uptake of [^18^F]FCH by aseptic chronic inflammation was consistent with lower uptake of [^18^F]FCH than that of [^18^F]FDG in aseptic chronic inflammation [20]. 

[^11^C]DMCYS, an analogue of S-[^11^C]methyl-L-cysteine ([^11^C]LMCYS) [23], which was recently developed by our research group, could be a novel amino acid transport tracer for tumor imaging. Like [^11^C]LMCYS, high uptake of [^11^C]DMCYS in tumors and low uptake in inflammatory tissue were observed (Supplemental Table 2). The possibly reason was that [^11^C]DMCYS, like [^11^C]LMCYS [23], could be also associated with the increased amino acid transport (such as the L system) and its lack of incorporation into protein. Thus, [^11^C]DMCYS could be a more specific PET tracer for tumor imaging than [^18^F]FDG.

Phosphatidylserine (PS) presented on the outer leaflet of apoptotic and necrotic neutrophils is ordinarily sequestered in the plasma membrane inner leaflet and provides itself as an excellent target for localizing inflammatory foci [24]. [^11^C]DPA-Zn^2+^ was developed as a novel PS-targeted apoptotic small-molecule tracer in our lab, with high uptake in apoptotic cells and almost no uptake in tumors [25]. It was reported that fluorescent DPA-Zn^2+^ analogues had a selective affinity for membrane enriched in anionic PS in the apoptotic cells [24] and have also been localized in infectious tissues induced with bacterial or nonbacterial pathways [24,26]. Furthermore, the higher accumulation of DPA-Zn^2+^ in bacteria inflammatory tissue than that in turpentine-induced inflammatory tissue owed to the more apoptotic neutrophils recruited due to the microbial infection [24,26]. In this work, we found mild uptake of [^11^C]DPA-Zn^2+^ in inflammatory tissue, which could be a result of the existence of less apoptotic neutrophils in turpentine-induced inflammatory tissue.

[^11^C]PIB is a *β*-amyloid-targeted PET tracer for Alzheimer’s disease (AD) imaging. AD is associated with *β*-amyloid plaques, neurofibrillary pathology, and neuronal damage. Neuro-inflammation is also a well-known feature of AD. However, it was reported that there was no uptake of [^11^C]PIB in neuroinflammation [12], which was similar to our result in that [^11^C]PIB exhibited almost no uptake in IM in this work. 

Though a clinical PET/CT scanner has its limitations, it can be quite adequate for sequential noninvasive imaging of small animals in research, because the CT is of high resolution, allowing for localization of PET findings and for more precise noninvasive estimation of radioactivity concentration through partial volume corrections [8]. In this work, we further confirmed the feasibility of using clinical PET/CT scanners to perform small animal model imaging. 

## 3. Experimental 

### 3.1. Generals

All reagents, unless otherwise specified, were of analytical grade and commercially available. All chemicals and solvents were purchased from Sigma-Aldrich (Milwaukee, WI, USA). Sep-Pak^©^ Plus C18 cartridges were obtained from Waters (Milford, MA, USA). The employed high performance liquid chromatography (HPLC) system (Alltima C18, Alltech, Lexington, KY, USA) was equipped a with UV detector (Alltech 201, USA) and radioactivity detector (Beijing PET Co. Ltd., Beijing, China), and radiochemical purities were checked by analytical HPLC. 

### 3.2. Radiosynthesis of PET Tracers

^11^CH_3_I was prepared from reduction of ^11^CO_2_ with LiAlH_4_, hydrolysis of the organometallic complex intermediate formed, and subsequent iodination of [^11^C]-methanol with hydrogen iodide. [^11^C]methyl triflate was produced from ^11^CH_3_I. [^11^C]DMCYS was synthesized according to the solid-phase [^11^C]-methylation of the precursor D-cysteine loaded into a C18 column with ^11^CH_3_I, similar to the production of [^11^C]LMCYS [23]. [^11^C]DPA-Zn^2+^ was synthesized by methylation of the precursor 2,2′-dipicolylamine (DPA2) with [^11^C]methyl triflate in acetone, hydrolysis with HCl for deprotection and chelation with zinc nitrate. [^11^C]PIB was produced from ^11^CH_3_OTf using a procedure similar to the reported method for the synthesis of ^11^C-labeled 2-β-carbomethoxy-3-β-(4-fluorophenyl)tropane ([^11^C]CFT) [27]. ^18^F^−^ was produced by the reported method [10] using ^18^O-water and proton bombardment on a cyclotron (Cyclone 10/5, IBA, Louvain-La-Neuve, Belgium). [^18^F]FDG was obtained from fully automated FDG synthesis module (IBA). [^18^F]FCH was synthesized using [^18^F]fluoromethyl triflate ([^18^F]CH_3_OTf) [11]. To determine radiochemical purity, analytical HPLC was performed using an Agilent 1200 Series HPLC system (Agilent Technologies, Sterling, VA, USA) equipped with a ZORBAX Eclipse XDB-C18 analytical column (4.6 × 150 mm, 5 μm; Agilent) using the different mobile phaseds at a flow rate of 1 mL/min. The elution profile was detected with an ultraviolet detector (Agilent interface 35900E, Agilent Technologies) at 254 nm (or 220 nm) and a B-FC-3200 high energy PMT Detector (Bioscan. Inc, Washington, DC, USA).

### 3.3. Inflammatory Mice Models

The study was performed according to the guidelines and recommendations of the Committee on Animal and Human Research at the First Affiliated Hospital, Sun Yat-Sen University. The protocol was fully approved by the local institutional review committee on animal care. Ninety female and male Kunming mice (6–8 weeks old, 20–25 g weight) were provided by the Experimental Animal Center, Sun Yat-Sen University. They were housed five animals *per* cage under standard laboratory conditions at 25 °C and 50% humidity. They were allowed free access to food and water. Turpentine oil (Beijing Chemical Reagent Co., Beijing, China, 0.20 mL) was inoculated into the mice right thigh muscle to induce inflammation [14]. The mass of the inflammatory tissue grew to 10–15 mm during the experiments within 3–4 days after the turpentine oil inoculation. Their success criteria were difficulty in walking or lame behavior. Ninety mice with stable and successfully indiced inflammation were randomly divided into six groups according to the different PET tracers at five different time points (5, 15, 30, 45, and 60 min) after injection of the different PET tracers above (three mice *per* group *per* time point).

### 3.4. PET/CT Imaging

PET imaging was performed using a Gemini GXL-16 scanner (Philips, Amsterdam, the Netherlands). Only the inflammatory mice in the 60 min post-injection groups (three mice per group) were taken for the PET image acquisition. Before the animals underwent [^18^F]FDG, [^18^F]FCH, ^18^F^−^, [^11^C]PIB, [^11^C]DPA-Zn^2+^, and [^11^C]DMCYS PET studies, they were kept fasting for at least 4 h. The inflammatory model mice were anesthetized with 10% chloral hydrate solution (3 mL/kg) before injection of the radiotracer and remained anesthetized throughout the study. The radiotracers (10 MBq) were injected in 200–300 µL phosphate-buffered saline or normal saline through the tail vein. Then, the mice were tested at 5 min after intravenous injection starting with a low-dose CT scan (30 mAs), immediately followed by a PET scan at 5, 15, 30, 45, and 60 min after injection of radiotracer. The PET scans were in 3-dimensional mode, with emission scans of 5–10 min *per* bed position. The CT scan was used for attenuation correction and localization of the lesion site. The PET and CT fused images were prepared using the vendor-supplied software of the scanning machine. Image evaluation was performed with small field of view (FOV) PET, CT, and fused PET/CT images. All PET images were reconstructed using an iterative reconstruction technique. The coronal, transaxial, and sagittal views of PET imaging in the model mice were obtained after image reconstruction with a slice thickness of 2.0 mm. 

### 3.5. Time-Course of Tracer Uptake Ratios

All the inflammatory mice in the six groups were sacrificed at different designated times. Blood (BL) was collected and main tissues including brain (BR), heart, lung, liver (LI), stomach, intestine, pancreas, kidney, femur, the right inflammatory thigh muscle (IM), and left normal non-inflammatory thigh muscle (NM) were excised. Moreover, the tissue samples were weighed and the radioactivity was determined with a gamma (γ) counter (GC-1200, USTC Chuangxin Co.Ltd. Zonkia Branch, Hefei, China). The accumulation of all tracers in the tissues was expressed as the percentage activity of injected dose per gram of tissue (%ID/g). The time-course (5, 15, 30, 45, and 60 min) of tracer uptake ratios for the IM/NM, IM/BL, IM/BR, and IM/LI was obtained. Among these time points, the 60 min-uptake ratio curves were acquired after PET imaging.

### 3.6. Histopathological Analysis

The inflammatory tissue and the normal muscle were fixed in 7.5% formaldehyde neutral buffer solution after their radioactivities were counted. The markedly swollen and pale tissues were dehydrated, embedded in paraffin, and sectioned at 2 μm thickness. Hematoxylin–eosin (HE) staining was performed for histopathological analysis. HE staining was used to assess endplate changes. The histopathological observation was performed using light microscopy. 

### 3.7. Statistical Analysis

Differences among the six groups were tested for statistical significance using Tamhane’s one-way ANOVA by SPSS (version 17.0) software assuming equal variances. *P* values of less than 0.05 were considered significant. Results were expressed as mean ± SD. 

## 4. Conclusions 

Consistent with biodistribution in mice with turpentine-induced inflammation, PET imaging shows that the uptake of ^18^F^−^ in IM is higher than that of [^18^F]FDG, whereas the uptakes of other PET tracers ([^18^F]FCH, [^11^C]DPA-Zn^2+^, [^11^C]DMCYS, and [^11^C]PIB) in IM are lower than those of [^18^F]FDG. [^18^F]FCH and [^11^C]DMCYS are potential PET tracers for tumor imaging, with an advantage of low tracer accumulation in IM over [^18^F]FDG. ^18^F^−^ seems to be a promising PET tracer for inflammation imaging. On the contrary, [^11^C]PIB is not good for the detection of inflammation due to its almost negligible radioactivity uptake in IM. As to [^11^C]DPA-Zn^2+^, it seems to be another potential PET tracer for inflammation imaging. Nevertheless, slightly low uptake of [^11^C]DPA-Zn^2+^ in inflammation requires us to further optimize its chemical structure. 

## Figures and Tables

**Table 1:** Uptake of [^18^F]FDG, ^18^F^−^, [^18^F]FCH, [^11^C]PIB, [^11^C]DPA-Zn^2+^, and [^11^C]DMCYS in inflammatory mice at 60 min after injection (%ID/g, n = 3 per group, Mean ± SD).

Tissue	[^18^F]FDG	^18^F^−^	[^18^F]FCH	[^11^C]PIB	[^11^C]DPA-Zn^2+^	[^11^C]DMCYS
Blood	1.36 ± 0.14	0.54 ± 0.12	1.17 ± 0.08	0.47 ± 0.08	1.36 ± 0.12	1.58 ± 0.52
Brain	4.99 ± 0.39	0.17 ± 0.02	0.30 ± 0.01	0.23 ± 0.02	1.27 ± 0.35	1.45 ± 0.48
Heart	12.5 ± 2.23	0.52 ± 0.03	2.50 ± 0.35	0.40 ± 0.11	0.61 ± 0.07	1.16 ± 0.68
Lung	2.2 ± 0.68	0.41 ± 0.05	3.11 ± 0.47	0.31 ± 0.05	0.66 ± 0.18	1.35 ± 0.73
Liver	1.88 ± 0.27	0.35 ± 0.02	20.51 ± 12.24	0.33 ± 0.04	3.31 ± 1.09	1.10 ± 0.28
Stomach	1.51 ± 0.31	0.28 ± 0.02	2.30 ± 0.31	0.30 ± 0.06	0.60 ± 0.14	0.91 ± 0.42
Pancreas	3.11 ± 0.38	0.41 ± 0.01	4.26 ± 0.67	0.39 ± 0.11	0.41 ± 0.06	4.17 ± 1.54
Kidney	3.89 ± 0.16	0.61 ± 0.05	8.00 ± 1.69	0.29 ± 0.05	1.29 ± 0.39	0.79 ± 0.35
Intestine	2.22 ± 0.34	0.39 ± 0.08	5.09 ± 0.72	0.33 ± 0.07	2.73 ± 0.85	1.08 ± 0.29
Femur	3.78 ± 2.53	21.97 ± 8.02	2.19 ± 0.25	0.06 ± 0.02	0.19 ± 0.03	0.12 ± 0.03
Muscle	1.48 ± 1.22	0.42 ± 0.04	0.97 ± 0.01	0.31 ± 0.04	0.83 ± 0.13	1.24 ± 0.46
Inflammation	4.33 ± 1.44	4.72 ± 0.70	1.78 ± 0.02	0.31 ± 0.03	1.69 ± 0.15	1.64 ± 0.11
IM/BL ^a^	3.18 ± 0.28	8.74 ± 1.58	1.52 ± 0.26	0.64 ± 0.13	1.24 ± 0.27	0.99 ± 0.18
IM/NM ^b^	2.93 ± 0.31	11.23 ± 2.32	1.83 ± 0.51	1.01 ± 0.09	1.73 ± 0.32	1.32 ± 0.25

^a^ The uptake ratio of radioactivity for inflammation to blood; ^b^ The uptake ratio of radioactivity for inflammation to normal muscle.

## Supplementary Materials

Supplementary materials can be accessed at: http://www.mdpi.com/1420-3049/17/12/13948/s1.
