# Supplementary material for: A Comparative Uptake Study of Multiplexed PET Tracers in Mice with Turpentine-Induced Inflammation
Source: Molecules. 2012 Nov 26;17(12):13948–59. doi: 10.3390/molecules171213948 (PMC6268432; doi:10.3390/molecules171213948)
Supplement: Supplementary file 1 [file molecules-17-13948-s001.pdf]

Supplementary Materials

Table S1. Time-uptake ratios of six PET tracers including IM/NM, IM/BL, IM/BR and IM/LI at different time (n = 3 per group, Mean ± SD).

**Tissue**	Tracers	**5 min**	**15 min**	**30 min**	**45 min**	**60 min**
IM/NM	[^{18}F]-FDG	1.39 ± 0.23	1.74 ± 0.42	2.14 ± 0.59	2.75 ± 0.67	2.81 ± 0.65
	$^{18}F^{-}$	1.11 ± 0.13	1.44 ± 0.32	3.48 ± 0.88	8.74 ± 1.09	10.98 ± 1.21
	[^{18}F]-FCH	1.21 ± 0.25	1.46 ± 0.27	1.58 ± 0.33	1.74 ± 0.38	1.83 ± 0.43
	[^{11}C]-PIB	0.93 ± 0.07	0.97 ± 0.09	0.98 ± 0.05	1.02 ± 0.07	1.07 ± 0.11
	[^{11}C]-DPA-Zn^{2+}	1.02 ± 0.08	1.13 ± 0.12	1.24 ± 0.15	1.44 ± 0.20	1.73 ± 0.13
	[^{11}C]-DMCYS	1.13 ± 0.20	1.33 ± 0.13	1.45 ± 0.21	1.78 ± 0.13	1.27 ± 0.09
IM/BL	[^{18}F]-FDG	0.98 ± 0.21	1.69 ± 0.32	2.31 ± 0.43	2.87 ± 0.26	3.63 ± 0.51
	$^{18}F^{-}$	0.14 ± 0.01	0.31 ± 0.04	2.80 ± 0.31	4.92 ± 0.51	8.17 ± 0.69
	[^{18}F]-FCH	5.29 ± 0.61	3.28 ± 0.43	1.73 ± 0.14	1.52 ± 0.09	1.45 ± 0.12
	[^{11}C]-PIB	0.86 ± 0.09	0.97 ± 0.06	1.19 ± 0.10	1.38 ± 0.14	1.57 ± 0.25
	[^{11}C]-DPA-Zn^{2+}	2.01 ± 0.36	1.32 ± 0.23	1.19 ± 0.12	1.05 ± 0.11	1.24 ± 0.27
	[^{11}C]-DMCYS	3.68 ± 0.33	2.6 ± 0.32	2.05 ± 0.24	1.58 ± 0.18	0.96 ± 0.09
IM/BR	[^{18}F]-FDG	0.32 ± 0.01	0.45 ± 0.04	0.56 ± 0.07	0.69 ± 0.08	0.78 ± 0.11
	$^{18}F^{-}$	14.23 ± 1.42	12.22 ± 1.09	8.32 ± 0.95	7.92 ± 0.54	25.64 ± 2.58
	[^{18}F]-FCH	8.32 ± 0.96	7.04 ± 0.89	5.33 ± 0.64	5.30 ± 0.54	5.27 ± 0.48
	[^{11}C]-PIB	0.88 ± 0.11	1.00 ± 0.14	1.07 ± 0.12	1.21 ± 0.23	1.32 ± 0.22
	[^{11}C]-DPA-Zn^{2+}	1.43 ± 0.28	1.32 ± 0.20	1.27 ± 0.17	1.09 ± 0.15	0.9 ± 0.06
	[^{11}C]-DMCYS	3.51 ± 0.38	2.33 ± 0.19	1.72 ± 0.12	1.32 ± 0.11	1.11 ± 0.07
IM/LI	[^{18}F]-FDG	0.12 ± 0.01	0.97 ± 0.10	1.28 ± 0.13	1.96 ± 0.21	2.12 ± 0.27
	$^{18}F^{-}$	0.89 ± 0.04	1.40 ± 0.21	2.77 ± 0.28	7.62 ± 0.62	12.76 ± 1.10
	[^{18}F]-FCH	0.12 ± 0.02	0.07 ± 0.01	0.08 ± 0.01	0.08 ± 0.01	0.08 ± 0.01
	[^{11}C]-PIB	0.70 ± 0.10	0.72 ± 0.08	0.80 ± 0.11	0.87 ± 0.13	0.91 ± 0.09
	[^{11}C]-DPA-Zn^{2+}	0.86 ± 0.11	0.73 ± 0.08	0.54 ± 0.06	0.38 ± 0.02	0.26 ± 0.03
	[^{11}C]-DMCYS	0.13 ± 0.01	0.56 ± 0.13	1.24 ± 0.22	1.65 ± 0.27	1.42 ± 0.28

Note: IM refers to inflammatory thigh muscle; NM refers to normal uninflammatory thigh muscle; BL refers to blood; BR refers to brain; and LI refers to liver.

Because [^{18}F]FCH and [^{11}C]DMCYS showed lower accumulation in inflammatory tissue than [^{18}F]FDG, we compared the target-to-nontarget ratios of [^{18}F]FDG, [^{18}F]FCH, [^{11}C]DMCYS in the S180 fibrofibrosarcoma-bearing and inflammatory mice (in the same animals) at 60 min after injection of the tracers (%ID/g, n = 3 per group), as shown in Supplemental Table 2.

Table S2. The target-to-nontarget ratios of [^{18}F]FDG, [^{18}F]FCH, [^{11}C]DMCYS in S180 fibrosarcoma-bearing and inflammatory mice at 60 min after injection (%ID/g, n = 3 per group, Mean ± SD).

**Tissue**	**[^{18}F]FDG**	**[^{18}F]FCH**	**[^{11}C]DMCYS**
IM/NM [a]	2.93 ± 0.31	1.83 ± 0.51	1.32 ± 0.25
T/NM [b]	5.60 ± 2.82	4.52 ± 1.68	8.43 ± 1.23

Notes: [a] The uptake ratio of radioactivity for inflammation to normal muscle; [b] The uptake ratio of radioactivity for tumor to normal muscle.

In Vitro Stability

Two samples of [^{11}C]DPA-Zn^{2+} and [^{11}C]DMCYS (1.48 MBq, 20 μL respectively) dissolved in normal saline were added to 200 μL of mice serum and incubated at 37 °C. A respective aliquot of the serum sample (20 μL) was injected into an HPLC column at 1 h to analyze their stability in mice serum. The experiment was performed using three separate samples for each tracer, respectively. According to the HPLC analysis, [^{11}C]DPA-Zn^{2+} and [^{11}C]DMCYS were proved to be stable *in vitro* and no degradation of the tracers was observed in mice serum at 37 ℃ for 1 h.

In Vivo Stability

Two female normal Kunming mice were anesthetized with 10% chloral hydrate solution (3 mL/kg) and injected with a dose of approximately 10.0 MBq (270 μCi) of [^{11}C]DPA-Zn^{2+} and [^{11}C]DMCYS, respectively, in 200 μL of normal saline via the tail vein. The mice were sacrificed at 20 min (for ^{11}C labeled compounds) after injection, and then the respective urine was carefully collected and filtered through a 0.22 μm Millipore filter into a tube. Respective aliquots of the urine sample (20 μL) were injected into an HPLC column to analyze the *in vivo* stability in the mice. Blood was obtained through the eyeball and centrifuged (6,000 rpm, 4 min), then filtered through a 0.22 μm Millipore filter to analyze by HPLC. In the *in vivo* stability, the radioactivities were approximately 20% of [^{11}C]DMCYS and 80% of its metabolic product in urine and most metabolites of [^{11}C]DMCYS existed in blood 20 min after injection. However, [^{11}C]DPA-Zn^{2+} was stable *in vivo* and no metabolites were found in urine or blood.
